# Effect of hydro-ethanol extract of *Caesalpinia pulcherrima (L.) Sw.* leaves in human and rat: *In vitro* approach of male contraceptive development

**DOI:** 10.5935/1518-0557.20240085

**Published:** 2025

**Authors:** Pampa Lohar, Dibya Pal, Tanusree Mondal, Puja Das, Debidas Ghosh

**Affiliations:** 1 Molecular Medicine, Nutrigenomics and Public Health Research Laboratory, Department of Bio-Medical Laboratory Science and Management, Vidyasagar University, Midnapore 721 102, West Bengal, India; 2 Centre for Life Sciences, Vidyasagar University, Midnapore 721 102, West Bengal, India

**Keywords:** In vitro, Caesalpinia pulcherrima, male contraceptive, spermiological sensors, oxidative stress

## Abstract

**Objective:**

The study focused the contraceptive efficacy of hydro-ethanolic (60:40) extract (HEE) of *Caesalpinia pulcherrima* leaves in human and rat sperm samples by *in vitro* study.

**Methods:**

Six young fertile adult males were selected for semen collection. Sperm samples were collected from six adult rat also by chopping the epididymis along with the collection of testicles, epididymis, and liver. The semen, sperm, and tissue samples were grouped into control, 1, 2, and 4 mg HEE exposed categories. Sensitive spermiological sensors, androgenic key enzymes, oxidative stress, and metabolic toxicity markers were assessed according to standard protocols. Human semen samples, rat sperm samples and metabolic tissue samples were divided into 16 test tubes in all of the above groups to find out the direct effect of the extract on such sensors in concentration and duration dependent manner.

**Results:**

Spermiological sensors both in human and rat were decreased significantly (*p*<0.05) in concentration and duration dependent manner after *in vitro* exposure of HEE against the control group. Testicular ∆5,3β-hydroxysteroid dehydrogenase, 17β-hydroxysteroid dehydrogenase, superoxide dismutase, and catalase activities were significantly (*p*<0.05) decreased, and level of the end product of lipid oxidation-peroxidation was elevated (*p*<0.05) in rat after extract charging. No general toxicity imposition of the said extract indicated by the activities of hepatic transaminases. Phytochemical screening was done by qualitative and liquid chromatography-mass spectrometry analysis.

**Conclusion:**

Extract focused the promising male contraceptive potentiality at 2 mg/ml concentration. Mode of action will be unfolded from running *in vivo* study very shortly.

## INTRODUCTION

Rapid population growth is one of the major issues in emerging nations like India. According to 2011 census of India, the population crossed 1.21 billion, and the world population will rise to 9.2 billion by the year 2050, which will impose a negative impact on social, economic, health, and natural resources (Shibeshi *et al*., 2006; Deeming, 2021). There is an urgent need to control the human population to ensure the betterment of human life. Family planning is an important strategy to control the overburdened population. Different contraceptives are used to tackle this global problem. Several female contraceptives are marketed, but males have not focused attentively in this regard, though responsibility for family planning is equal for male and female spouses (Verma & Yadav, 2021). So, now investigators have concentrated their investigation on male contraceptive regimens. In the market, several male contraceptives are procurable, though having high failure rates, and are not widely accepted. Different accessible steroidal contraceptives have severe side effects or toxic actions when used for short-term and long-term purposes (Jain *et al*., 2017). In that context, there is a need to establish a safe, efficient, eco-friendly, and affordable male contraceptive to slow down the increasing population. From ancient history, many plants have been adopted as a folk medicine for curative purposes of numerous diseases as well as for contraceptive purposes. Few herbs are familiar with having antifertility properties either by suppressing spermatogenesis or spermicidal action (Kamal *et al*., 2003).

This *in vitro* experiment was conducted to check the direct effect of the hydro-ethanolic (3:2) extract (HEE) of the *Caesalpinia pulcherrima* leaves on human and rat samples.

In consonance with this herbal plant-based study, previously our laboratory conducted a comprehensive study about the spermicidal effects of HEE of *Stephania hernandifolia* and *Achyranthes aspera* in rat and human sperm (Paul *et al*., 2010). According to *in vitro* studies of others, the compound NIM-76, which was extracted from neem oil, reported 100 percent spermicidal action against rat and human sperm (Riar *et al*., 1990). The seed extract of *Carica papaya* has an immobilizing effect on sperm noted by an *in vitro* study (Lohiya *et al*., 2000). *C. pulcherrima* also known as ‘Pride of Barbados’ locally known as “Radhachura,” belongs to the Fabaceae family, distributed mainly in West Bengal, Tamilnadu, Kerala, Karnataka, and throughout India. This plant has some impressive health benefits, found to possess antitumor, antimicrobial, abortifacient, cardioprotective, lipid-lowering, hepato-protective, antiulcer, antiasthma, and hypoglycemic activities (Mitra & Mukherjee, 2009; Kumar *et al*., 2010; Zanin *et al*., 2012). According to a traditional Chinese herbal medicine, *C. pulcherrima* has a folk reputation for possessing an antimotility effect on sperm (Pankaj *et al*., 2011). However, there is a lacuna of detailed information about the anti-spermiological activity of *C. pulcherrima* leaves. In our pilot study, HEE reflected the maximum effect for anti-testicular activity among other extracts. Therefore, HEE was selected as an effective extract in this concern. Till now, the mode of action for male contraceptive activity of this plant is beyond our knowledge. Considering this background, the present study was designed to explore the effect of the said plant on sperm parameters following an *in vitro* protocol with the goal of developing a potent herbal male contraceptive.

## MATERIALS AND METHODS

### Chemicals

Ethanol (Changshu Hongsheng Fine Chemical Co., Ltd, No.8 Haifeng Road, Changshu City, China) for extraction. Chemicals like glutaraldehyde, eosin, gelatin, and glycerol were also procured from Merck Life Science Pvt. Ltd. Bengaluru, Karnataka, India. Fructose, EDTA, were provided by Sisco Research Laboratories Pvt. Ltd., Maharashtra, India.

Hydrogen peroxide (H_2_O_2_) and sodium citrate were purchased from Merck, Mumbai, India. Pyrogallol was supplied by Loba Chemie Pvt. Ltd. Maharashtra, India.

### Collection of plant materials

*C. pulcherrima* mature leaves were collected from the Vidyasagar University campus in West Bengal, India. The leaves were cleaned using redistilled water and then dried under shade at room temperature and authenticated (No. VU/BIO/CP-2022) by a taxonomist in the Department of Botany and Forestry, Vidyasagar University. Leaves were ground into a coarse powder using an electric grinder.

### Plant extract preparation

Dried powder of *C. pulcherrima* leaves (100 g) was mixed with hydro-ethanol at the ratio 3:2 in 1 lit solvent, left for 48 hrs at room temperature and stirred at 2 hrs intervals. After that, the mixture was filtered. The solvent extract was evaporated using a rotary evaporator (EYELA, China). The remaining extract was allowed to dry, and this powdered extract was collected and kept at 4^º^C for this *in vitro* experiment.

### Experimental design for *in vitro* study

The biological samples of both the human and rat models were used to conduct this *in vitro* study. Human semen samples were obtained from 6 healthy fertile individuals after 4-5 days of sexual abstinence, and everybody gave their signed consent (Chaudhury *et al*., 2004). *In vitro* testing of the collected semen samples was performed after spontaneous coagulation and reliquification. The samples have normal sperm count, motility, viability, and normal morphology, as per World Health Organization (WHO) laboratory manual (World Health Organization, 2010).

In a rat model, regarding the care of animals, the standard protocol of the Institutional Ethics Committee (IEC) was followed for processing and handling of the samples. Six matured, fertile rats, two months of age and weighing 150 g, were selected for these investigations. For the acclimatization of rats in laboratory conditions, animals were kept for seven days in the controlled environment of the animal house. After that, the animals were sacrificed, and sperm cells were obtained by washing the cauda epididymis with normal saline. Testicles, epididymis, and liver were dissected and cleaned with normal saline. Insertions have been given in both poles of these organs for the proper penetration of *in vitro* media and extract. Sperm pellets (rat and human) were prepared using the centrifugation method. Krebs Ringer Bicarbonate (KRB) solution (pH 7.4) was used as an *in vitro* medium.

Focusing the statistical standpoint, here we increased the sample size 6 to 16 by cutting the tissues or distributing the sperm samples of 6 subjects to 16 for noting the authentic effect of the extract. Simultaneously from the ethical point of view, 6 animals were used in each group or sub group.

Here, experimental groups were divided into the following-

### Experimental groups

#### Control groups

Human and rat sperm samples, sliced tissues such as testicles, epididymis, and liver of rat were kept in sixteen separate test tubes for each sample (n=16) with 10 ml KRB solution to maintain the osmotic balance and optimal pH of the medium without any extract exposure. Sperms of rat and human were mixed with physiological saline in a 1:1 ratio. Then, at 37^º^C, the mixture was incubated, maintaining 95% O_2_ and 5% CO_2_ supply for two hrs at a velocity of 30 bubbles per min. The spermiological sensors were investigated at the intervals of 20 secs, 15 mins, and 30 mins incubation periods. Sperm pellets (rat and human) and the above-mentioned tissues were kept in test tubes without any extract exposure for biochemical assessment at the end of the incubation period (2 hrs) considered as control group.

#### HEE - charged groups

Test tubes contained 10 ml KRB media, where prementioned extract at the concentration of 1, 2, and 4 mg/ ml were directly charged in different test tubes and kept in an incubator. For each dose exposed group, sixteen test tubes were allotted for each sample. Separate test tubes were used for human and rat samples study.

### Sperm motility

Motile sperm were counted and expressed in terms of percentage in control and three concentrations of HEE (1, 2, 4 mg/ml) charged groups for separate three duration of exposure. A drop of the incubated *in vitro* mixture from the control and duration dependent different extract-charged groups was put on different glass slides, covered by a coverslip, and viewed under 400X magnification (Olympus, Olympus Opto Systems India Pvt. Ltd. Noida, India) to evaluate sperm motility (Zemjanis, 1970).

### IC_50_ value of sperm motility

Fifty percent inhibitory concentration of (IC_50_) the extract on sperm motility was calculated for both human and rat (Ratnasooriya *et al*., 1991).

### Sperm viability

The viable sperm were determined, taking no color by eosin and counter-stained by nigrosine. The pink-stained (dead) and unstained (living) sperm on the prepared slides were counted under a 400X microscope and noted in terms of percentage (World Health Organization, 1999).

### Hypoosmotic swelling (HOS) test

The structural and functional integrities of the plasma membrane of spermatozoa were evaluated by the exposure of the sperm cells to a hypo-osmotic solution following a standard method (Jeyendran *et al*., 1984). The suspension of sperm was combined with pre-warmed solution (0.735% of sodium citrate and 1.351% of fructose in 100 ml distilled water) at the ratio of 1:9 and incubated at 37^º^C for 2 hrs. Tail curling was observed to indicate the normal integrity of spermatozoa through microscopic observation and expressed in percentage.

### Acrosomal intactness status (AIS) test

Gelatin was used to coat the clean glass slide, and after 24 hrs, it was fixed with 0.05 % glutaraldehyde solution to assess the acrosomal status of sperm. Smears were made on gelatin-coated slides using diluted sperm samples. The gelatin was broken down by sperm-derived enzymes, which make haloes surrounding the head part of sperm, and the percentage was recorded (Gopalkrishnan, 1995).

### Estimation of activities of ∆5,3β-hydroxysteroid dehydrogenase (HSD) and 17β-HSD

Androgenic key enzymes, i.e., ∆5,3β-HSD and 17β-HSD, activities were assessed (Jarabak *et al*., 1962; Talalay, 1962). The solution contains five mM of potassium phosphate, 20% spectroscopic graded glycerol, and 1 mM EDTA. The testicular sample was allowed for homogenization (Bio-Lab, India) at a tissue density of 1g/10ml, allowing for centrifugation (HERMLE, Germany) for 30 mins at 10000 rpm at 4^º^C. The enzyme kinetics was measured using the supernatant as per the standard methods. At intervals of 30 secs, optical density at 340 nm was recorded for 3 mins using a spectrophotometer (Thermo Fisher Scientific, China).

### Estimation of antioxidant enzyme activities

Testis, epididymis, and liver tissues from rats, as well as sperm pellets from both rat and human samples, were homogenized in an ice-cold Tris-HCl buffer (0.05 M) at a tissue concentration of 50 mg/ml for the assessment of superoxide dismutase (SOD) and catalase (CAT) activities. Then, the supernatant was collected by centrifugation (4^º^C) at 10000 rpm for 10 mins.

In a cuvette, 2.04 ml (50 mM) Tris buffer (pH-8.2), 20 µl of pyrogallol, and 20 µl of the sample were added for the SOD activity assessment. Optical density was noted in each sample in comparison to a blank at 420 nm in 30 secs intervals for three mins (Marklund & Marklund, 1974).

In a spectrophotometer cuvette, 0.5 ml of 0.00035 M H_2_O_2_ (Merck, Mumbai, India), 2.5 ml of distilled water, and 40 µl of the sample were added. After that, at every 30 secs interval, six readings of the sample against the blank were collected at 240 nm to estimate CAT activity (Beers & Sizer, 1952).

### Estimation of thiobarbituric acid reactive substances (TBARS)

A well-acceptable method was used for the assessment of TBARS level. Testicular tissue, epididymis, and liver from rat whereas sperm pellets of human and rat were homogenized in 0.1 M of ice-cold phosphate buffer (pH 7.4) at a 50 mg/ml tissue concentration and centrifuged at 10000 rpm at 4^º^C for 5 mins. The optical density was noted at 535 nm in a spectrophotometer (Ohkawa *et al*., 1979).

### Assessment of aspartate aminotransferase (AST) and alanine aminotransferase (ALT) activities

The activities of AST and ALT were also measured in the liver, testis, and epididymis after *in vitro* exposure of the extract in the said concentrations of the rat tissue samples using the standard methods (Jagadeesan & Kavitha, 2006). Two reaction mixtures were prepared separately, one for AST and another for ALT. After one hr for AST and 30 mins for ALT, the reaction mixtures were incubated at 37^º^C after adding 0.2 ml homogenate. The reaction was terminated by the addition of 1 ml DNPH reagent, and the test tubes were maintained at room temperature for twenty mins. The color was developed by introducing 10 ml of 0.4N sodium hydroxide solution, and optical density was recorded at 520 nm using the UV spectrophotometer against blank.

### Phytochemical investigation of HEE of *C. pulcherrima* leaves

The presence of phytochemicals, i.e., alkaloids, flavonoids, tannins, phenolics, saponins, terpenoids, glycosides, and steroids in the decoction of *C. pulcherrima* leaves were studied qualitatively using the standard methods (Mitra *et al*., 2020).

### Liquid chromatography-mass spectrometry (LCMS) analysis

The QuattroMicro^TM^API mass spectrometer and Waters 2695 separation module (Waters, Milford, MA, USA) were used to perform LC-MS studies. The liquid chromatographic system comprised the quaternary pump, autosampler, online vacuum degasser, and thermostatic column compartment. It was linked in line to a photodiode array detector (Waters 2998) in front of the mass spectrometer. MassLynx 4.1 software (Waters) was considered for noting data and processing. The autosampler injected a 10 µl sample (HEE of *C. pulcherrima* leaves) into the LC system. Solvent ‘A’ was 0.1% aqueous formic acid, and solvent ‘B’ was 6% methanol: acetonitrile (2:1), used as a mobile phase. Using a PDA detector, every compound was found to be between 191-690 nm (Das *et al*., 2023).

### Ethical considerations

This study was ethically permitted from Institutional Animal Ethics Committee (VU/IAEC/10/7/2022). Informed consent was taken from each of the participant as per human ethics protocol of ICMR.

### Statistical analysis

The results were presented as mean±standard error of the mean (SEM). ANOVA followed by “Multiple-comparisons Student’s two-tail - ‘t’ test” was used for statistical analysis of data (Sokal & Rohle, 1997).

## RESULTS

### Sperm motility

In both human and rat sperm, after exposure of different concentrations of extract, the percentage of motile sperm was decreased significantly (*p*<0.05) than the control group. According to the WHO (2010), more than 40% of motile sperm in semen is one of the determinants of male fertility. In human sperm samples, after exposure to 2 mg extract for ½ an hr, the number of motile sperm was noted below the WHO reference value. In the 1 mg HEE-charged group, less than 40% motile sperm was not observed in 20 secs, 15, and 30 mins incubation periods. In the 4 mg HEE-charged group, the reference level of WHO value of motile sperms for infertility was noted at all incubation times, and after 30 mins of incubation, complete immobilization in human sperm was observed.

**Figure 1:**
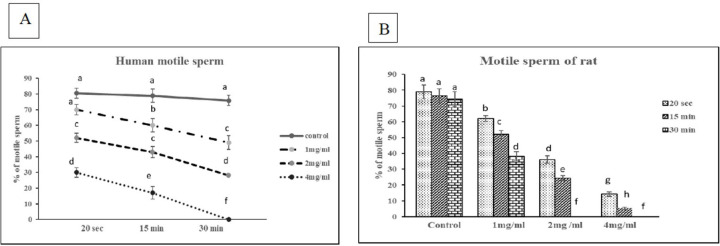
Concentration and duration dependent effects of HEE (60:40) of *C. pulcherrima* leaves on the percentage of motile sperm in (A) human and (B) rat samples followed by different incubation times (20 secs, 15 and 30 mins). Points and bars were expressed as mean± SEM (n=16). ANOVA followed by “Multiple-comparison Student’s two-tail ‘t’-test” was performed. Points and bars with different superscripts (a-h) differ from each other significantly, *p*<0.05.

As there are no such reference values for rat sperm analysis, the WHO reference value of human was translated for rat sperm analysis. Less than 40% motile sperm were noted in the 1 mg/ml extract-exposed group after 30 mins of incubation. However, in 2 and 4 mg/ml extractcharged groups, the motile sperm count was less than the borderline of the said reference value in all said incubation times. After 30 mins of incubation, both 2 and 4 mg HEEcharged groups showed a 100 % immobilization effect on rat sperm (Figure 1).

### IC _50_ value of sperm motility

**Figure 2:**
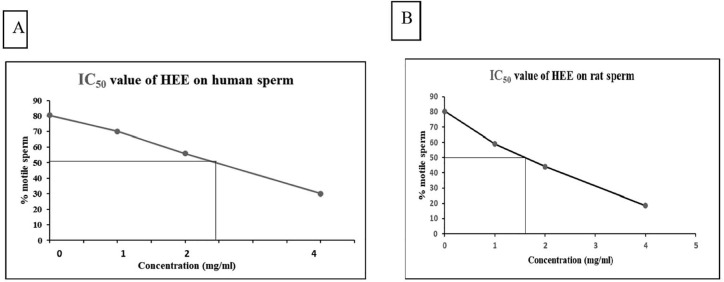
Determination of IC_50_ value of HEE (60:40) of *C. pulcherrima* leaves on (A) human and (B) rat sperm motility.

The concentration of the extract results for 50% inhibition in the count of motile sperm, known as IC _50_, and the value of the above-said extract for human sperm was noted at 2.45 mg/ml concentration, and for rat sperm, it was 1.6 mg/ml (Figure 2).

### Sperm viability

In both human and rat sperm, after being exposed to different concentrations of extract, the percentage of viable sperm was significantly lower (*p*<0.05) than the control group. According to WHO (2010), less than 58% of viable sperm results infertility. After direct exposure to the different concentrations of extract to human spermatozoa, less than 58% viable sperm were observed after 30 mins of incubation in the 2 mg HEE-charged group. In contrast, in the 1 mg HEE-charged groups, the viable sperm count percentage remained above the lower borderline in all the above three durations of exposure. In the 4 mg HEEcharged groups, the viable sperm percentage was below the reference value in all three incubation periods.

**Figure 3:**
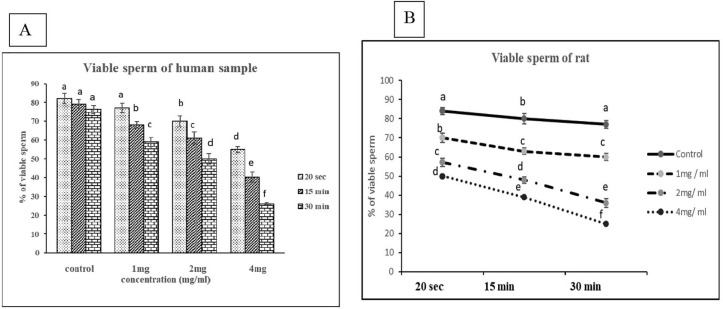
The direct effect of HEE of *C. pulcherrima* leaves on the percentage of viable sperm of (A) human and (B) rat in concentration and duration-dependent manner. Bars and points were expressed as mean±SEM (n=16). ANOVA followed by “Multiple-comparison Student’s two-tail ‘t’-test.” Bars and points with different superscripts (a-f) differ from each other significantly, *p*<0.05.

In case of rat, viable sperm count was below the WHO reference value at 2 and 4 mg/ml HEE-charged groups after all the said incubation times. However, less than 58% of viable sperms was not observed in 1 mg HEE-charged groups in all said durations of exposure (Figure 3).

### Hypoosmotic swelling (HOS) test

According to WHO, less than 58% of swelled human sperm indicated as abnormal semen and results infertility. After direct exposure to said plant extract at different concentrations (1, 2, 4 mg/ml), hypoosmotic swelled tail curling sperm (human and rat) were significantly decreased (*p*<0.05) when compared to the control. In human spermatozoa, at 2 mg concentration, the HOS sperm were below the WHO cut-off value after 15 and 30 mins of incubation, whereas, in the concentration of 4 mg, the value was noted in all three incubation periods. This sensor was not below that limit at the 1 mg HEE-charged groups at any above three incubation periods.

**Figure 4:**
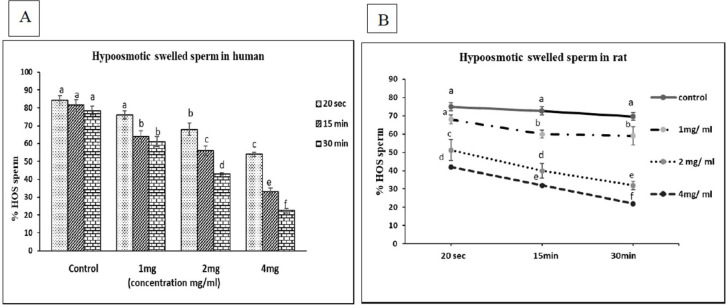
The direct effect of HEE of *C. pulcherrima* leaves on the percentage of hypoosmotic swelled sperm of (A) human and (B) rat at different concentrations for different incubation periods. Bars and points were expressed as mean±SEM (n=16). ANOVA followed by “Multiple-comparison Student’s two-tail ‘t’test.” Bars and points with different superscripts (a-f) differ from each other significantly, *p*<0.05.

Less than 58% of hypoosmotic swelled sperm of rats were present in all the said three durations when it was exposed to 2 and 4 mg/ml HEE of *C. pulcherrima* leaves. In contrast, after the 1 mg HEE-charging, the percentage of HOS-positive sperm below the cut-off level was not noted in any duration of said exposure (Figure 4).

### Acrosomal intactness status (AIS) test

**Figure 5:**
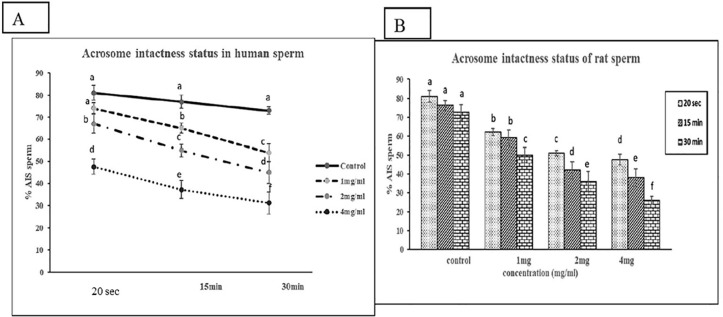
Effect of HEE of leaves of *C. pulcherrima* on the percentage of sperm with acrosomal intactness status of (A) human and (B) rat for different incubation periods at different concentrations. Bars were expressed as mean±SEM (n=16), ANOVA followed by “Multiple-comparison Student’s two-tail ‘t’-test”. Points and bars with different superscripts (a-f) differ from each other significantly, *p*<0.05.

Direct exposure of sperm to different concentrations of *C. pulcherrima* leaves extract showed a significant (*p*<0.05) reduction in acrosome intact spermatozoa count with respect to the control. In human, less than 40% of sperm with intact acrosomes are reflected as a lower limit for fertility (Chan *et al*., 1999). After 15 and 30 mins of incubations with direct exposure to 4 mg HEE, the human sperm with intact acrosome count were below the reference value. However, the value of these sensors remained above that reference value in all the said incubation periods in 1 and 2 mg HEE-charged groups. In rat spermatozoa, below the stated cut-off value was noted at 2 mg HEEcharged group after 30 mins of incubation, and similarly, at 4 mg HEE-charged group, less than the cut-off value with intact acrosome were noted after 15 and 30 mins of incubation time. However, the count remained above that reference value in all the said durations at 1 mg HEEexposed groups (Figure 5).

### Activities of testicular ∆5, 3β- HSD and 17β-HSD

After two hrs of incubation, testicular ∆5,3β- HSD, and 17β-HSD activities were significantly lower (*p*<0.05) in the groups subjected for exposure to 2 and 4 mg doses than the group exposed to 1 mg dose and the control groups. Significant alteration was not noted in these parameters between the control and the group subject to 1 mg exposure.

**Figure 6:**
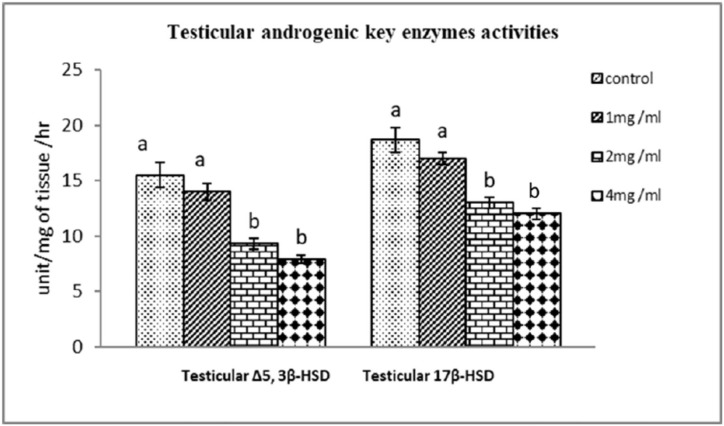
Direct effect of HEE of *C. pulcherrima* leaves on testicular androgenic key enzyme activities at different concentrations in rat. Bars were expressed as mean±SEM (n=16). ANOVA followed by “Multiplecomparison Student’s two-tail ‘t’-test.” Bars with different superscripts (a-b) differ from each other significantly, *p*<0.05.

In comparison between 2 and 4 mg/ml charged groups, the HEE of *C. pulcherrima* showed a statistically insignificant difference (*p*>0.05) of the above parameters (Figure 6).

### Estimation of antioxidant enzyme activities

After 2 hrs of incubation, the SOD and CAT activities in the testis and epididymis of experimental rats were significantly inhibited (*p*<0.05) in 2 and 4 mg HEE-charged groups compared to the control group. However, significant differences (*p*>0.05) were not noted in the activities of these parameters between 1 mg and the control groups.

Activities of SOD and CAT in rat sperm pellets were inhibited significantly (*p*<0.05) in all extract-exposed groups in respect to the control. But in the human sperm pellets, SOD and CAT activities were reduced significantly (*p*<0.05) in 2 and 4 mg extract exposed groups, whereas no significant (*p*>0.05) inhibition was observed between 1 mg and the control groups. The activities of said sensors in hepatic tissue were not altered significantly (*p*>0.05) among the groups subjected to extract exposure and the control group (Table 1).

**Table 1:** Effect of different doses of HEE of C. *pulcherrima* on the activities of SOD and CAT in the testis, epididymis, sperm pellet and liver after 2 hrs of incubation. Data expressed as mean±SEM, n = 16. Values in columns with different superscripts (a-c) differ from each other significantly; p<0.05. ANOVA followed by "Multiplecomparison Student's two-tail 't'-test. Values in parenthesis indicate the percentage of diminution (;) in respect to the control.

Experimental groups	Anti-oxidative enzymes									
	SOD (unit/mg of tissue)					CAT (pM of H_2_O_2_ consumed / mg of tissue/min)				
	Reproductive tissue		Sperm pellet (Rat)	Sperm pellet (Human)	Metabolic tissue (liver) (Rat)	Reproductive tissue		Sperm pellet (Rat)	Sperm pellet (Human)	Metabolic tissue (liver) (Rat)
	Testis (Rat)	Epididymis (Rat)				Testis (Rat)	Epididymis (Rat)			
Control	1.44±0.07^a^	1.55±0.06^a^	2.51±0.19^a^	9.67±0.31^a^	2.16±0.05^a^	2.56±0.12^a^	1.86±0.09^a^	4.58±0.05^a^	38.57±1.80^a^	8.23±0.71^a^
1 mg HEE- exposure group	1.32±0.02^a^ (8.33%↓)	1.42±0.02^a^ (8.38%↓)	1.42±0.09^b^ (43.42%↓)	8.59±0.54^a^ (11.16%↓)	1.98±0.06^a^ (8.33%↓)	2.41±0.07^a^ (5.85%↓)	1.82±0.09^a^ (2.15%↓)	3.16±0.05^b^ (31%↓)	37.44±1.31^a^ (2.92%↓)	7.63±0.51^a^ (7.29%↓)
2 mg HEE- exposure group	0.88±0.03^b^ (38.88%↓)	0.93±0.04^b^ (40%↓)	0.92±0.07^b^ (63.34%↓)	6.55±0.37^b^ (32.26%↓)	1.85±0.18^a^ (14.35%↓)	1.98±0.06^b^ (22.65%↓)	1.4±0.09^b^ (24.73%↓)	2.34±0.03^c^ (48.90%↓)	27±1.11^b^ (29.99%↓)	7.12±0.78^a^ (13.48%↓)
4 mg HEE- exposure group	0.82±0.02^b^ (43.05%↓)	0.88±0.03^b^ (43.22%↓)	0.85±0.07^b^ (66.13%↓)	5.01±0.21^c^ (48.19%↓)	1.88±0.11^a^ (12.96%↓)	1.88±0.06^b^ (26.56%↓)	1.33±0.08^b^ (30.10%↓)	2.25±0.09^c^ (50.87%↓)	21±0.97^c^ (45.55%↓)	6.98±0.96^a^ (15.18%↓)

### Quantity of thiobarbituric acid reactive substances (TBARS)

After 2 hrs of incubation in 2 and 4 mg HEE-exposed groups, the level of TBARS was significantly elevated (*p*<0.05) in the reproductive tissue sample, i.e., testis and epididymis of rat when comparison was made with the control group. No significant difference was noted in TBARS level (*p*>0.05) between 1 mg and the control groups.

The quantity of TBARS was elevated significantly (*p*<0.05) in rat sperm pellets in all the groups exposed to different doses compared to the control. In human sperm pellets, the level of TBARS was significantly increased (*p*<0.05) in 2 and 4 mg extract exposed groups with respect to the control and 1 mg exposed groups.

Insignificant changes (*p*>0.05) in hepatic TBARS level were noted among the exposed charged and control groups (Table 2).

**Table 2:** Effect of different concentrations of HEE of *C. pulcherrima* on the level of TBARS in testis, epididymis, sperm pellet and liver after 2 hrs of incubation. Data expressed as mean±SEM, n=16. Values in each column with different superscripts (a-c) differs from each other significantly; *p*<0.05. ANOVA followed by “Multiple-comparison Student’s two-tail ‘t’-test". Values in parenthesis indicate the percentage of elevation (↑) in respect to the control.

Experimental groups	Free radical end products				
	TBARS (mM/ mg of tissue)				
	Reproductive tissue		Sperm pellet (Rat)	Sperm pellet (Human)	Metabolic tissue (liver) (Rat)
	Testis (Rat)	Epididymis (Rat)			
Control	17.49±1.18^a^	15.88±1^a^	27.66±0.95^a^	32.32±1.41^a^	145±1.92^a^
1 mg HEE- exposure group	18.71±1.13^a^ (6.97% ↑)	16.48±1.9^a^ (3.77% ↑)	35.34±1.32^b^ (27.76%↑)	34.60±1.36^a^ (7.05% ↑)	148±1.89^a^ (2.06%↑)
2 mg HEEexposure group	25.80±0.08^b^ (47.51% ↑)	23.92±1.51^b^ (50.62%↑)	47±1.74 ^c^ (69.92%↑)	39.07±1.54^b^ (20.88%↑)	150±1.88^a^ (3.44%↑)
4 mg HEEexposure group	26.11±0.09^b^ (49.28%↑)	24.64±1.76^b^ (55.16%↑)	49.12±1.48^c^ (77.58%↑)	50.32±1.55^c^ (55.69%↑)	157±0.81^a^ (8.27%↑)

### Assessment of aspartate aminotransferase (AST) and alanine aminotransferase (ALT) activities

Activities of AST and ALT in the liver, testis, and epididymis in the rat showed no significant difference (*p*>0.05) in all HEE-exposed groups when a comparison was made with the control group (Table 3).

**Table 3:** Effect of different doses of HEE of *C. pulcherrima* leaves on AST and ALT activities in the liver, testis, and epididymis in albino rat after 2 hrs incubation. Computed values were expressed as mean±SEM, n=16. “Multiple-comparison Student’s two-tail ‘t’-test” after ANOVA was used for data analysis, Values in each column with same superscript (a) did not differ from each other significantly; *p*>0.05.

	AST activity (unit/mg of tissue)			ALT activity (unit/mg of tissue)		
**Organs** **Groups**	**Liver**	**Testis**	**Epididymis**	**Liver**	**Testis**	**Epididymis**
Control	25.08±0.59^a^	20.76±0.72^a^	22.2±0.35^a^	21.68±0.28^a^	21.08±0.77^a^	24.19±0.27^a^
1mg extract exposure	25.47±0.64^a^	21.81±0.78^a^	22.74±0.38^a^	21.97±0.26^a^	21.87±0.70^a^	24.58±0.24^a^
2 mg extract exposure	25.80±0.63^a^	22.67±0.73^a^	23.19±0.34^a^	22.33±0.29^a^	22.77±0.67^a^	24.72±0.23^a^
3 mg extract exposure	23.13±0.60^a^	23.11±0.77^a^	23.28±0.32^a^	22.46±0.27^a^	23.40±0.71^a^	24.95±0.26^a^

### Analysis of the phytochemicals of HEE of *C. pulcherrima* leaves

Biochemical analysis of the nature of phytomolecule

(s) showed the presence of alkaloids, flavonoids, tannins, and terpenoids in the HEE of *C. pulcherrima* leaves (Table 4).

**Table 4:** Phytochemical constituents of HEE of C. pulcherrima leaves. (-, absent; +, moderate level present; ++, high level present).

Phytomolecule(s)				Methods			Appearance	
Alkaloid				Dragendorff’s test Hager’s test			**++** **+**	
Flavonoid				Ferric chloride test			**++**	
Tannin				Ferric chloride test			**+**	
Terpenoid				Salkowski’s test			**++**	
Saponin				Foam test			-	
Phenol				Ferric chloride test			-	
Glycoside				Molisch test			-	

### Liquid chromatography-mass spectrometry (LC-MS) analysis

**Figure 7:**
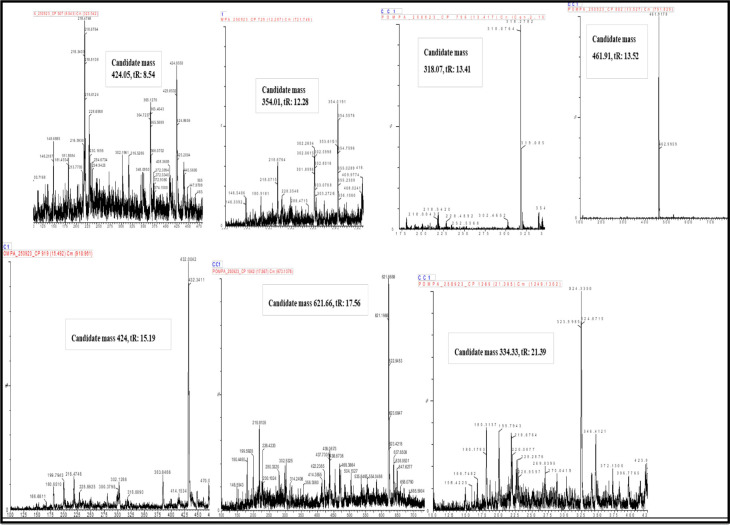
Seven compounds of HEE of *C. pulcherrima* leaves with candidate mass (m/z).

The LC-MS analysis of HEE of *C. pulcherrima* leaves showed seven major peaks with retention times of 8.54, 12.28, 13.41, 13.52, 15.49, 17.56, and 21.39 mins. Subsequently, every peak underwent fragmentation, given seven fragmentation spectra featuring potential masses (m/z) at positive ions 424.05, 354.01, 318.23, 432, 621.66, and 324.33 and one at negative ions with having candidates mass (m/z) 461.91 (Table 5, Figure 7).

**Table 5:** Identified compounds with retention time (RT) of HEE of *C. pulcherrima* leaves by LC-MS analysis based on literature evidence.

Sl. no.	RT (Min)	Ionization Mode	Observed m/z		Reference m/z	Name of the proposed compound		Nature of the compound	
1	8.54	+	424.05		424.37	Amyrone		Terpenoid	
2	12.28	+	354.01		354.13	Protopine		Alkaloid	
3	13.41	+	318.27		318.23	Myricetin		Flavonoid	
4	13.52	-	461.91		461	Chrysoeriol hexoside		Phenol	
5	15.49	+	432.00		432.37	Apigenin diglycosides		Flavonoid	
6	17.56	+	621.66		621	Apigenin glucoside		Flavones	
7	21.39	+	324.33		324.12	Dimethylene berberine		Alkaloid	

## DISCUSSION

Human and rat sperm were used in this *in vitro* experiment to determine the impact of the HEE of *C. pulcherrima* leaves on sperm-disrupting activity in relation to the development of male contraceptive. Rat sperm, reproductive, and metabolic tissues and human semen samples were included to unfold the functioning principle of the phytomolecule(s) that existed in the extract for the execution of male contraceptive effects. As clear cutoff values are not available for routine sperm analysis of rat and their fertility assessment like human so, we have used the WHO reference values of spermiological sensors of human for the analysis of spermiological parameters of rat in relation to validate the male contraceptive efficacy of the extract. For this purpose, the cut-off values for the fertility of human sperm were translated into rat. Sperm motility is a vital sensor for male fertility assessment. Sperm fertilizing capacity depends not only on its motility but also on sperm membrane intactness and acrosome status. The motility of spermatozoa is depends on the generation of adenosine triphosphate (ATP) via oxidative phosphorylation (Tourmente *et al*., 2015). A considerable amount of decrement in sperm motility percentage in the extract-charged group, possibly due to the disruption of sperm mitochondrial function and inhibition in ATP generation by phytomolecule(s) (Riar *et al*., 1990). It was supported by the IC_50_ value for sperm motility of the HEE of *C. pulcherrima* leaves. A lower percentage of viable and hypoosmotic swelled sperm (human and rat) after extract exposure, that affect the membrane integrity possibly by imposition of lipid peroxidation along with the spermplasma membrane destruction by inducing oxidative stress (Agarwal *et al*., 2014). These have been confirmed by the elevation in the quantity of free radical end products, i.e., TBARS level in reproductive tissues (testis, epididymis) and sperm pellets (human and rat), as well as decreased activities of antioxidant enzymes, i.e., SOD and CAT in reproductive tissues of rat (testis, epididymis) and sperm pellets (human and rat). The said extract may have a sperm-specific effect supported by less oxidative stress imposition in secondary reproductive and metabolic organs. This may be due to the high content of saturated fatty acids in sperm cells (Agarwal *et al*., 2014). It is generally recognized that the plant derivatives severely impede the sperm cells membrane-related enzymes hyaluronidase and acrosin, which are required for the sperm cell to proceed forward in the cervix to fertilize (Sabeti *et al*., 2016). After extract exposure, inactivation of these acrosomal enzymes may occur by the phytomolecule(s), which results in low interaction with gelatine-coated slides. The extract may ultimately hamper the capacitation and acrosome reactions of sperm necessary for fertilization (Dutta *et al*., 2021). The reduction in two key steroidogenic enzyme activities, i.e., ∆5, 3β-HSD, and 17β-HSD activities, also supports the contraceptive efficacy of the extract. This inhibition possibly due to the presence of phytomolecule(s) that act as competitive or non-competitive inhibitor(s) by binding with the enzymes or by altering the structure of the enzymes through allosteric modulation (Wang *et al*., 2016). The preliminary phytochemical investigation of HEE extract of *C. pulcherrima* leaves revealed the presence of alkaloid, flavonoid, terpenoid, and phenol. The LCMS analysis of the plant extract also confirms the three flavonoids (myricetin, apigenin diglycosides, and apigenin glucoside), two alkaloids (protopine, and dimethylene berberine), one phenol (chrysoeriol hexoside) and one terpenoid (amyrone). According to existing literature, the presence of these phytochemical components resulted spermicidal, antispermatogenic, and antiandrogenic activities (Verma & Yadav, 2021). So, it may be expressed that the HEE of *C. pulcherrima* leaves has a negative modulatory effect on sperm characteristics such as motility, viability, acrosome cap status, and HOS in a concentrationdependent manner. Activities of AST and ALT in the liver, testis, and epididymis were measured in this experiment to identify the reproductive and metabolic toxicity of HEE of *C. pulcherrima* leaves. Insignificant changes in the level of such parameters reflected that different concentrations of leaf extract used here don’t have any toxic effects on both reproductive and metabolic organs, which focused that the said extract at the above doses may be used safely.

In human and rat sperm, 2 mg concentration showed the maximum therapeutic effect on spermiological sensors and other concerning parameters, whereas 1 mg of HEE showed no such effectiveness for spermicidal effect in all the cases. Though 4 mg in a few cases is more effective than 2 mg dose, from the viewpoint of safety factors, 2 mg dose is considered as optimal for this purpose. One possible hypothesis is that the extract may contain the requisite amount of the concerned phytomolecule(s) in 2 mg HEE that results in maximum efficacy for this purpose in most of the sensors compared to other doses (Ghosh *et al*., 2018). No additional therapeutic response was shown at 4 mg concentration, which may be explained by spare receptor theory or receptor reserve or efficiency reserve theory, which provides a buffer enabling the phytomolecule(s) to maintain their efficacy even if not all receptors are occupied at any given time. The efficiency of the 2 mg dose is not identical in both human and rat, which may be due to species-specific activity.

Despite the promising findings from our *in vitro* experiments on the HEE of *C. pulcherrima* leaves for male contraceptive development, this study still has some limitations need to be addressed. *In vitro* experiments, although valuable for initial screening and mechanistic studies, may not fully replicate the complex physiological environment of the body which is focused by *in vivo*. Factors such as metabolism, systemic circulation, and tissue-specific interactions are not captured *in vitro*. This limitation underscores the need for subsequent *in vivo* studies to confirm the efficacy and safety of the extract in a more physiologically relevant context (Ghallab & Bolt, 2014).

## CONCLUSION

From this *in vitro* experiment, it may be concluded that the HEE of *C. pulcherrima* leaves has direct male contraceptive activity addressing some limitations though future research is essential.
